# The Effectiveness of the Buzzy Device for Pain Relief in Children During Intravenous Injection: Quasirandomized Study

**DOI:** 10.2196/15757

**Published:** 2022-04-29

**Authors:** Yen-Hua Cho, Yi-Chien Chiang, Tsung-Lan Chu, Chi-Wen Chang, Chun-Chu Chang, Hsiu-Min Tsai

**Affiliations:** 1 Pediatric Department Linkou Chang Gung Memorial Hospital Taoyuan City Taiwan; 2 School of Nursing College of Medicine Chang Gung University Taoyuan City Taiwan; 3 School of Nursing Chang Gung University of Science and Technology Taoyuan City Taiwan; 4 Division of Pediatric Hematology and Oncology Linkou Chang Gung Memorial Hospital Taoyuan Taiwan; 5 Administration Center of Quality Management Department Chang Gung Medical Foundation Taoyuan City Taiwan; 6 Division of Pediatric Endocrinology and Genetics Department of Pediatrics LinKou Chang Gung Memorial Hospital Taoyuan City Taiwan; 7 Department of Nursing Linkou Chang Gung Memorial Hospital Taoyuan City Taiwan

**Keywords:** hospitalized children, intravenous injection, pain, Buzzy

## Abstract

**Background:**

Intravenous injection is the most common medical treatment and the main cause of pain in hospitalized children. If there is no appropriate health care for pain relief, the proportion of moderate and severe pain often exceeds 70%. With nonpharmaceutical-based pain management, Buzzy is recognized as an effective device for rapidly relieving injection pain in hospitalized children. However, Buzzy is not widely used in Asia and very few experimental studies in Asia have addressed the effectiveness of the Buzzy device at treating needle pain in hospitalized children.

**Objective:**

The main purpose of this study was to investigate the effectiveness of the Buzzy device for diminishing pain levels among hospitalized children in Taiwan.

**Methods:**

We applied a quasiexperimental design with random assignment. According to the time of admission, child participants were randomly assigned to treatment and nontreatment groups. The Buzzy device was applied as an intervention in this study. The samples size was 30 per group. The study participants were recruited from the pediatric ward of a medical center in northern Taiwan. The research data were collected longitudinally at three time points: before, during, and after intravenous injection. Three instruments were used for assessment: a demographic information sheet, the Wong-Baker Face Scale (WBFS), and the Faces Legs Activity Cry Consolability (FLACC) scale. The data were analyzed by descriptive analysis, the Mann-Whitney *U* test, the Wilcoxon signed-rank test, and the *χ*^2^ test.

**Results:**

A total of 60 hospitalized children aged 3 to 7 years participated in this study, including 30 participants in the treatment group and 30 participants in the nontreatment group. The average age of children in the treatment and nontreatment groups was 5.04 years and 4.38 years, respectively. Buzzy significantly mitigated pain in children during intravenous injection with a significant difference between the two groups in pain-related response (FLACC) and actual pain (WBFS) (*Z*=*–*3.551, *P*<.001 and *Z*=–3.880, *P*<.001, respectively). The children in the treatment group had a significantly more pleasant experience than those in the nontreatment group (*Z*=–2.387, *P*=.02). When Buzzy was employed, the children experienced less pain than they did during previous intravenous injections (Z=–3.643, *P*<.001).

**Conclusions:**

The intervention of using the Buzzy device was effective in reducing pain levels of intravenous injection among hospitalized children. The specific focus on children in Asia makes a valuable contribution to the literature. For clinical application, the reliable pain relief measure of Buzzy can be used in other Asian children to help health care providers improve noninvasive care among children. For future applications, researchers could integrate Buzzy into therapy-related games and a technology-based app to increase the efficiency of use and provide more data collection functions.

## Introduction

### Background

Hospitalization is an extremely stressful process for children, who may exhibit degenerative or aggressive behavior when undergoing medical treatments such as intravenous injections. The related literature indicates that intravenous injection is the most common medical treatment and the main cause of pain and fear in hospitalized children [1-3]. Intravenous injection often causes stress to both children and nurses, resulting in difficulty of the cannulation process and a higher likelihood of multiple attempts being required. Researchers reported that administering an intravenous injection to hospitalized children is highly difficult; two or more attempts were needed in 67.3% of cases [4,5]. The average number of attempts for intravenous insertion was found to be 4.2 [2]. During repeated attempts, a child experiences moderate or severe pain, with the pain rating reaching up to 71.0%-79.6% [6,7]. This not only wastes medical supplies and nursing time but also heightens the tension and affects the trust relationship between nurses and hospitalized children [8,9]. With respect to other long-term impacts, researchers have found that 62.3% of children fear the pain caused by injection and 62.9% have negative memories of injection [8], resulting in a negative experience that affects their behavioral response to pain during future invasive treatments [10-12]. Therefore, it is crucial for nurses and health care providers to effectively relieve children’s pain during injections and to mitigate their fear as well as to affect a positive experience of injection [13].

With nonpharmaceutical-based pain management, Buzzy is recognized as an effective device to be used for rapidly relieving injection pain in hospitalized children. Through a cooling sensation and vibration, Buzzy is easy use, inexpensive, and fast-acting for reducing procedural pain [14-16]. Buzzy does not require substantial preparation time before the injection and provides effective pain relief. Buzzy is increasingly used during various medical procedures, including intravenous injections [12,14,17,18], the drawing of blood [15,19-22], and vaccination [23,24]. Buzzy successfully mitigates treatment-related pain, fear, and anxiety in pediatric patients. Whelan et al [20] discovered that Buzzy not only relieved pain in children but that 80% of children further wished to use the device during their next injection.

Numerous benefits of the Buzzy device have been reported, such as its short preparation time and ease of use, along with benefits of the cute design in distracting children to reduce pain and fear during injections [17,23,25]. However, Buzzy is not widely used in Asia. According to a literature review, there are very few experimental studies in Asia to address the effectiveness of the Buzzy device at treating needle pain in hospitalized children [26]. No clinical study has been reported in applying the Buzzy device in hospitalized children in Taiwan. Hence, in this study, we used Buzzy during intravenous injections in hospitalized children and determined its effectiveness at pain relief. Empirical data from this study can provide clinical evidence to understand the effectiveness of Buzzy devices for the clinical work environment in regions of Asia. Given the specific focus on children in Asia, this study should make a valuable contribution to the literature for researchers and health care providers.

### Research Purpose and Hypotheses

The main purpose of this study was to investigate the effectiveness of the Buzzy device for diminishing pain levels among hospitalized children in Taiwan. The specific aims of the study were to determine (1) the pain levels of pediatric patients during intravenous injections, (2) the effectiveness of Buzzy for pain relief during intravenous injection in pediatric patients, (3) relevant factors that affect the effectiveness of Buzzy, (4) the degree of influence that injection experience has on needle pain in preschool children during intravenous injection, and (5) the demographics of children with different pain levels during intravenous injection.

According to the purpose of the study, the following six research hypotheses were tested:

1. The pain level of pediatric patients is lower when Buzzy is used during intravenous injection.
2. The actual pain experienced by those given intravenous injection using Buzzy is lower than the expected pain.
3. Intravenous injection in pediatric patients takes less time when Buzzy is employed.
4. More pediatric patients report a satisfactory experience with intravenous injection when Buzzy is employed.
5. If a child has had an unpleasant injection experience in the past, their pain level during the present injection will be higher.
6. The younger the child, the higher the pain level will be during intravenous injection; the pain level during intravenous injection is higher for girls than boys.

## Methods

### Study Design

This was a quantitative study with a quasiexperimental design. Patient participants were alternately assigned to each group upon admission. According to their time of admission and order of recruitment, the children were randomly assigned to the treatment or nontreatment group. For example, the first child to arrive was assigned to the treatment group and the second child to arrive was assigned to the nontreatment group. Each case was assigned a number to protect patient privacy.

The research data were collected longitudinally. The treatment and nontreatment group data were collected at three time points: before, during, and after intravenous injection. Before injection, a questionnaire and interview were used to understand the injection experience of the participant. The Wong-Baker Face Scale (WBFS) was used to measure the expected pain of the children before their intravenous injection. The Face, Legs, Activity, Cry, Consolability (FLACC) scale was used to measure the behavioral response of the children to pain during intravenous injection. The WBFS was used after the injection to measure the actual pain felt by the children during intravenous injection. In the treatment group, the Buzzy device was placed on the participant 5 cm above the intravenous site before needle insertion.

### Participants

The inclusion criteria of the study were as follows: (1) age 3-7 years, with the child accompanied by their parent; (2) required to receive intravenous injection during hospitalization; (3) could speak Mandarin or Taiwanese with clear consciousness; and (4) participation consent of the patient or parent(s). The exclusion criteria were (1) cognitive or developmental delay or an inability to speak clearly, (2) chronic disease, (3) operation required because of external injury or inflammatory condition, and (4) refusal to complete the pain assessment scales and resistance to measurement of vital signs.

The estimated number of participants for this study was calculated according to the previous study of Moadad et al [12] and the method of estimating sample sizes in two-group comparisons [27]. Based on previous clinical research of the Buzzy device for pain management, Moadad et al [12] indicated that a total sample size of 50 was acceptable. With a power of 0.8, an acceptable two-sided 5% significance level, and a difference of *d*=0.07, the sample size required per group in the two-group comparison was calculated to be 33 [27]. According to this previous research and considering the potential case turnover rate, the samples size was determined to be 40 per group in this study.

### Framework

**Figure 1:**
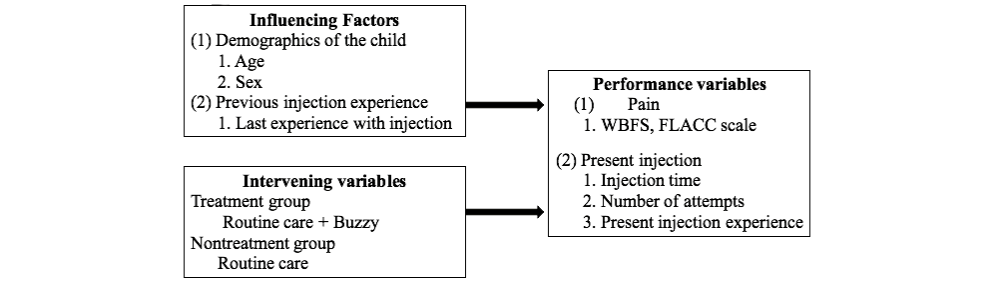
Study framework. FLACC: Faces, Legs, Activity, Cry, Consolability; WBFS: Wong-Baker Face Scale.

On the basis of the research purpose and results of a literature review, the conceptual framework of the study displayed in Figure 1 was proposed to elucidate the effects of Buzzy on the pain felt by children aged 3-7 years during intravenous injection. The pain level was then related to demographics and injection experience.

### Study Instruments

#### Overview

The research tools employed in this study were demographic information analysis, a questionnaire regarding injection experience, the WBFS and FLACC scale for assessing pain level, and Buzzy.

#### Demographic Data

The demographic information and injection experience questionnaire collected data on the basic information of the child (age, sex, and education level), the accompanying primary caregiver, previous injection experience (number of hospitalizations and last injection experience), present injection experience (injection duration and number of attempts), and experience and feelings about the present injection.

#### Pain Scales

##### Wong-Baker Face Scale

The WBFS [28] is a face scale displaying six cartoon faces depicting, from left to right, no pain to the highest pain, with respective pain scores of 0, 2, 4, 6, 8, and 10. The recruiter would point with their finger at the leftmost face and move rightward, explaining to the child that the faces toward the right indicate “more painful.” They asked the children to point to the face that best described their pain and the recruiter recorded the corresponding score. The pain scores were classified into mild pain (0-3), moderate pain (4-6), and severe pain (7-10). The reliability and validity of the WBFS have been confirmed by expert scholars, and the scale has reliable construct validity, convergent validity, and predictive validity. The Cronbach α is .82-.92 [29,30].

##### FLACC Scale

Using the FLACC scale [31], the recruiter observed the behavior of the children during intravenous injection: their facial expression, leg movement, activity, crying, and consolability. The child was assigned a score ranging from 0 to 2 for each behavior type, and the five scores were summed and recorded. A total score of 0 denoted that the child was relaxed and comfortable, a score of 1-3 denoted slight discomfort, a score of 4-6 denoted moderate discomfort, and a score of 7-10 denoted severe discomfort.

#### Buzzy Device

**Figure 2:**
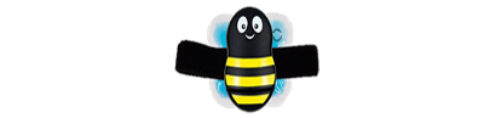
Image of the Buzzy device.

Buzzy is a device developed by the emergency pediatrician Amy Baxter, MD, in 2011. Buzzy is mainly based on the gate control theory of pain, aiming at the effect of cold and vibration at the injection site to achieve pain relief. The Buzzy device is approximately 8×5×2.5 cm in size, and its exterior design is in the shape of a bee (Figure 2). A thin ice bag resembling a wing is attached to the bottom of the main body of the device, which can be directly fixed above the injection site. Turning on the Buzzy device within 30 seconds to 1 minute before injection can significantly improve the pain of injection. The Buzzy device has demonstrated clear effects on pain and can be used for needle-related treatments, including intravenous injection, preventive injection, and blood draws [17,21,23,32].

In the treatment group, the recruiter secured Buzzy on the participant at a location 5 cm above the site of intravenous injection. The device was placed as close as possible to the site without affecting the injection process and results. The Buzzy device was switched on 1 minute before the injection and was turned off after completion of the injection.

### Data Collection

The recruiter explained the research purpose, methods, and content to the ward head nurse and nursing staff. The recruitment of participants and data collection were performed without affecting nursing care activities. Two nurses with 3 years of nursing experience were recruited for this study and participated in the study briefing workshop so as to understand the purpose of the study and the steps to perform it.

**Figure 3:**
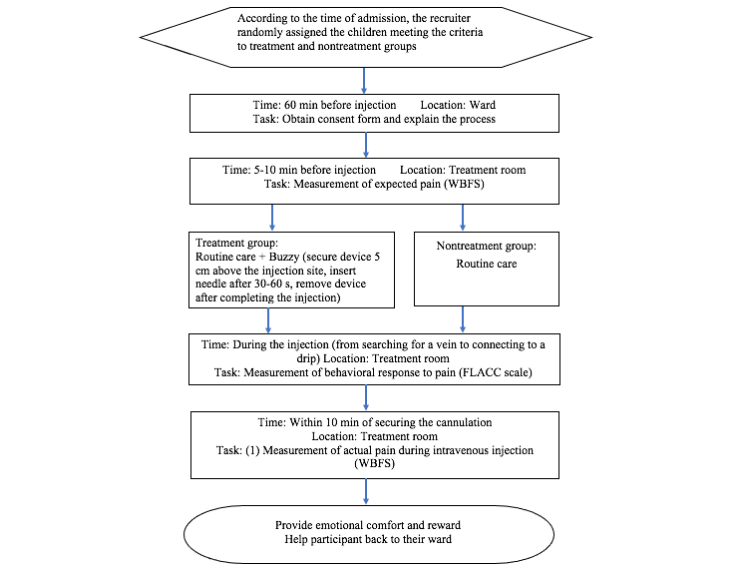
Flowchart of the study procedure. FLACC: Faces, Legs, Activity, Cry, Consolability; WBFS: Wong-Baker Face Scale.

The recruiters invited children that met our inclusion criteria to participate in the study and randomly assigned them to the treatment or nontreatment group according to their time of admission and order of recruitment. The recruiters explained the research purpose and method to the children and their caregiver(s), and after obtaining consent, the caregiver was asked to sign a consent form. For the treatment group, the recruiter explained the Buzzy device to the caregiver and child using a video and the Buzzy device. The data collection procedure is illustrated in Figure 3.

In the treatment group, the implementation of the intervention consisted of three main steps. First, a video was used to explain the Buzzy device and its operating procedures to the caregivers and the hospitalized children, while allowing the children to touch the Buzzy device and experience its vibration and coldness. Second, when the child came to the treatment room, the nurse and the recruiter fixed the Buzzy device with a belt to 5 cm above the injection site of the patient. Third, the Buzzy device was turned on within 1 minute before the injection so that the child could be attracted by the vibration and coldness of the device. The recruiter turned off the device when the nurse completed the injection.

### Data Analysis

We used SPSS Statistics version 22.0 for statistical analysis. The collected data were organized, assigned serial numbers, and then input to a computer system. According to the nature of the research variables, descriptive statistics (frequency distribution, mean and SD, and percentage) were obtained. The normality of the data in this study was checked before applying inferential statistical analysis. Tests for normality showed a nonnormal distribution of pain scales (WBFS, FLACC), duration, number of attempts, and previous and current intravenous experience. Therefore, group comparisons of these data were performed using the *χ^2^* test, Mann-Whitney *U* test, Wilcoxon signed-rank test, and Kruskal-Wallis test. This study used two-tailed tests with significance set at *P*<.05.

### Ethics Approval

This study program obtained prior approval from the Chang Gung Medical Foundation Institutional Review Board (201701889A3).

## Results

### Participant Characteristics

Our research participants were children aged 3-7 years that required intravenous injection during hospitalization. The recruitment period was January 30 to May 10, 2018, during which 64 children who met our criteria were invited to participate. Four primary caregivers declined. A total of 60 children/caregivers agreed to participate. According to their time of admission and order of recruitment, the participants were randomly assigned to the treatment or nontreatment group. Each group comprised 30 participants.

The average age of the treatment and nontreatment group was 5.04 years and 4.38 years, respectively. The treatment group had more male participants (19/30, 63%), whereas the nontreatment group had more female participants (18/30, 60%). In the treatment group, 24 participants (80%) had previously been hospitalized, whereas only 19 (63%) participants in the nontreatment group had previously been hospitalized. In the treatment group, 19 of the 30 participants (63%) had a previous unpleasant experience with intravenous injection, and 19 of 23 participants (83%) in the nontreatment group reported a previous unpleasant experience. The average duration of injection of the treatment and nontreatment groups was 6.63 and 6.57 minutes, respectively, which was not significantly different (*χ^2^_2_*=3.42, *P*=.18). The injection was successful at the first attempt for most children in both groups: 26/30 (87%) in the treatment group and 23/30 (77%) in the nontreatment group. Two attempts were required for the remaining children and more than two attempts were not required for any participant. The *χ^2^* tests of demographic information revealed no significant intergroup differences (*P*>.05) regarding age, sex, hospitalization experience, last injection experience, injection duration, and number of attempts, as detailed in Table 1.

**Table 1:** Demographic information of research participants (N=60).

Variable			Treatment group (n=30), n (%)		Nontreatment group (n= 30), n (%)		*χ* ^2^			*df*			*P* value	
**Age (years)**								7.38			3			.06
	3-4	5 (17)		13 (43)										
	4-5	6 (20)		7 (23)										
	5-6	14 (47)		9 (30)										
	6-7	5 (17)		1 (3)										
**Sex**								3.27			1			.07
	Male	19 (63)		12 (40)										
	Female	11 (37)		18 (60)										
**Inpatient experience**								2.05			1			.15
	No	6 (20)		11 (37)										
	Yes	24 (80)		19 (63)										
**Previous experience of intravenous injection**								2.38			1			.12
	Good (3-5)	11 (37)		4 (17)										
	Poor (0-2)	19 (63)		19 (83)										
**Injection duration (minutes)**								3.42			2			.18
	<5	20 (67)		16 (53)										
	5-10	4 (13)		10 (33)										
	>10	6 (20)		4 (13)										
**Number of attempts**								1.00			1			.31
	1	26 (87)		23 (77)										
	2	4 (13)		7 (23)										

### Level of Pain Relief With Buzzy

#### Expected Pain Before Employing Buzzy During Intravenous Injection

In the treatment room before injection, the WBFS was used to determine the pain that the children were expecting from the intravenous injection. The median pain score was 6.00 in the treatment group. Moderate pain, with a score of 4-6, was indicated by 46.67% (n=14) of the children, and severe pain, with a score of 7-10, was indicated by 36.67% (n=11) of the children; thus, overall, 83.34% of the children were expecting moderate or severe pain. The Mann-Whitney *U* test revealed no significant difference between the groups regarding the expected pain, as shown in Table 2.

**Table 2:** Comparison of pain scores between the treatment and nontreatment groups (N=60).

Variable	Treatment group (n=30), median	Nontreatment group (n=30), median	*Z*	*P* value
Expected pain before injection (WBFS^a^)	6.00	6.00	–0.659	.51
Behavioral response to pain during injection (FLACC^b^)	4.00	6.00	–3.551	<.001
Actual pain after injection (WBFS)	2.00	8.00	–3.880	<.001

^a^WBFS: Wong-Baker Face Scale.

^b^FLACC: Faces, Legs, Activity, Cry Consolability.

#### Pain-Related Response When Using Buzzy During Injection

Pain during injection denoted the pain experienced by the children from applying the tourniquet until needle insertion was completed and the drip was connected. During injection, the FLACC scale was used to score the behavioral responses of the children to pain. The median pain score was 4.00 in the treatment group; overall, 37% (n=11) of the children experienced moderate pain (score of 4-6) and 20% (n=6) experienced severe pain (score of 7-10). Thus, 57% (n=17) of the children experienced moderate or severe pain, which was 40% lower than the percentage in the nontreatment group. In the treatment group, 43% more children reported a pain score of less than 4 (mild pain) in comparison with the nontreatment group. The Mann-Whitney *U* test showed a significant difference between the two groups in behavioral responses to pain during injection (Table 2). Thus, Buzzy significantly ameliorated the children’s behavioral response to pain during injection.

#### Actual Pain Felt and Reported After Using Buzzy During Intravenous Injection

In the treatment room after injection, the WBFS was used to measure the actual pain felt by the children during the intravenous injection. The median pain score was 2.00 in the treatment group; 27% (n=8) of the children experienced moderate pain and 17% (n=5) experienced severe pain. Overall, 43% experienced moderate or severe pain, which was 47% lower than that in the nontreatment group. The Mann-Whitney *U* test revealed that the pain score after injection was significantly different between the two groups (Table 2). Thus, Buzzy significantly mitigated pain during intravenous injection.

#### Comparison of Expected Pain With Actual Pain When Buzzy Was Employed

The median expected pain before intravenous injection, determined using the WBFS, was 6.00 in the treatment group; 83% (n=25) of children reported a pain score of 4 or greater. The median actual pain score, reported after the intravenous injection and again using the WBFS, was 2.00 in the treatment group and 43% (n=13) of children reported a pain score of 4 or greater. The Wilcoxon signed-rank test showed a significant difference between expected and actual pain in the treatment group but not in the nontreatment group (Table 3). When Buzzy was employed, the actual pain was lower than the expected pain, confirming that Buzzy reduced the pain experienced during intravenous injection in children.

**Table 3:** Comparison of expected and actual pain (Wong-Baker Face Scale) in the two groups (N=60).

Group	Expected pain, median	Actual pain, median	*Z*	*P* value
Treatment group (n=30)	6.00	2.00	–2.652	.008
Nontreatment group (n=30)	6.00	8.00	–1.689	.09

#### Duration of Intravenous Injection, Number of Attempts, and Injection Experience When Buzzy Was Employed

The median duration of the injection procedure was 5 minutes in both the treatment and nontreatment groups, with no significant difference (Table 4). Thus, use of Buzzy did not lengthen the duration of the injection procedure. The median number of attempts was 1.00 in both the treatment and nontreatment groups, with no significant difference (Table 4). Therefore, use of Buzzy did not significantly affect the number of attempts at needle insertion.

The children were asked to rate their present intravenous injection experience using a rating scale of 0-5: extremely poor (0), very poor (1), poor (2), satisfactory (3), good (4), and excellent (5). The children in the treatment group rated their experience with the present intravenous injection significantly higher than that of children in the nontreatment group (Table 4). Therefore, the use of Buzzy provided pain relief during the injection and resulted in a less painful experience compared with that experienced when the device was not used.

**Table 4:** Table4. Comparison of duration, number of attempts, and previous and present intravenous injection experiences (N=60).

Variable	Treatment group (n=30), median	Nontreatment group (n=30), median	*Z*	*P* value
Injection duration (minutes)	5.00	5.00	–1.393	.16
Number of attempts	1.00	1.00	–0.993	.32
Previous intravenous injection experience^a^	1.50	1.00	–0.996	.32
Present intravenous injection experience^a^	4.00	2.50	–2.387	.02

^a^Rated on a scale of 0-5 from “extremely poor” to “excellent.”

### Degree of Influence of Injection Experience on Needle Pain

The interview and asked the children about their previous experiences of intravenous injection. After their injection, the children were requested to rate their experience with the injection on the previously mentioned scale of 0 (extremely poor) to 5 (excellent). The Mann-Whitney *U* test was used to determine the difference between the groups regarding their previous and present intravenous injection experiences. Regarding previous intravenous injection experiences, no significant difference was discovered between the groups (Table 4).

However, the use of Buzzy in the present intravenous injection gave the treatment group a less painful experience than the nontreatment group, representing a significant difference (Table 4). The Wilcoxon signed-rank test was applied to compare the experience of the previous and present injection within the groups, showing that both groups experienced less pain in the present intravenous injection than in previous intravenous injections (*Z*=–3.643, *P*<.001; Z=–2.348, *P*=.02). In summary, both groups had mostly negative previous experiences with intravenous injection, and the Buzzy device not only mitigated pain during the injection but further improved the children’s experience of the injection, generating positive experiences.

### Demographic (Sex and Age) Effects on Pain Level

The children were divided into four age groups: 3-4, 4-5, 5-6, and 6-7 years. Age group–based differences in experience with the present intravenous injection, expected pain before the injection, pain-related response during the injection, and actual pain felt were analyzed. The Kruskal-Wallis test revealed no significant differences between age groups regarding these variables (*P*>.05).

The Mann-Whitney *U* test was employed to determine the differences in experience between the sexes regarding their present intravenous injection, pain-related response during the injection, and actual pain felt. In the nontreatment group, experience with the present intravenous injection and pain-related response during the injection were significantly different between the sexes (*Z*=–2.441, *P*=.02; Z=–2.566, *P*=.01); however, no sex-based differences were significant in the treatment group.

## Discussion

### Effectiveness of Buzzy at Pain Relief

According to the results of Moadad et al [12] and Canbulat et al [17], the use of Buzzy significantly mitigates pain in children during intravenous injection. Our study obtained similar findings, with the two groups having significantly different (*Z*=–3.551, *P*<.001; *Z*=–3.880, *P*<.001) pain-related responses and actual pain. Our findings are consistent with those of previous studies, showing that Buzzy reduces needle pain in children. Additionally, Lin et al [10] revealed that the expected pain before intravenous injection predicted the pain level felt during an intravenous injection with 63.4% of the variance explained. In our study, the average expected and actual pain scores in the nontreatment group were greater than 6, indicating that the children’s expectations of pain were met; that is, the actual and expected pain were not significantly different (*Z*=–1.689, *P*=.09). However, when Buzzy was employed, the actual pain level was lower than the expected pain level (*Z*=–2.652, *P*=.008), revealing that Buzzy reduced needle pain in the children during intravenous injection.

In summary, in the absence of an effective intervention measure, the children experienced moderate or severe needle pain during intravenous injection, whereas when Buzzy was used, the behavioral response to pain during injection (FLACC score) and actual pain felt were significantly lower. We also discovered that although the purpose and operation of Buzzy had been explained to the children, because they had not previously seen or used Buzzy, the children remained anxious about needle pain, and therefore the expected pain was not significantly different between the two groups. During injection when Buzzy was employed, the children were more cooperative during the injection process; these children also reported less pain and a significant difference was achieved in comparison with that of the nontreatment group. The children also wished to use the device during their next injection.

### Degree of Influence of Buzzy on the Present Injection

Moadad et al [12] reported that the duration of intravenous injection did not differ between their treatment and nontreatment groups. The injection duration in this study was defined as the time from applying the tourniquet until the intravenous tube was connected to the drip. Buzzy was switched on 15 seconds to 1 minute before the injection was initiated and was switched off after the injection was complete. The entire procedure took less than 7 minutes for both groups, and the duration did not significantly differ between the groups (Z=–1.393, *P*=.16). This can be attributed to the injection being successful at the first attempt in most of the children, regardless of group (treatment group: 87%; nontreatment group: 77%). The intergroup difference in the rate of successful injection was nonsignificant (*Z*=0.993, *P*=.32). We thus found that using Buzzy did not affect the rate of successful injection or injection duration; however, the children in the treatment group were more cooperative during the injection process. This could increase the willingness of clinical staff to use Buzzy.

### Degree of Influence of Intravenous Injection Experience on Needle Pain

Hsieh et al [8] reported that 62.9% of children had an unpleasant experience of injection in the past. In our study, 19 children in both the treatment (63%, 19/30) and nontreatment (83%, 19/23) groups had a previous unpleasant experience, showing that most of the child participants had experience of intravenous injection and most of these experiences were unpleasant.

The literature suggests that hospitalized children have the ability to expect pain during injections, and combined with their previous experiences, each injection affects their attitude and feelings toward the next injection. This experience also affects their response to painful treatment in the future, and according to unpleasant previous experiences, the children have the same emotions and some may even resist treatment [10,33]. The children in our treatment group had a significantly more pleasant experience than those in the nontreatment group (*Z*=–2.387, *P*=.02). When Buzzy was employed, the children experienced less pain than they did during previous intravenous injections (*Z*=–3.643, *P*<.001). These findings indicate that a reliable pain relief measure should be used when administering intravenous injection to children to prevent an unpleasant experience from affecting their next injection. The pediatric wards of medical centers should thus use pain relief measures and consider including them as part of routine nursing procedures.

### Age- and Sex-Based Differences in Pain Levels

Karakaya and Gozen [30] reported that the particular age of preschool children did not affect their pain levels. However, some studies discovered that older children experience less pain (*P*=.03) [18] and younger children self-report stronger pain [10,12,34]. When an effective intervention was employed for pain relief, no significant age- and sex-based differences were discovered in one study [22]. In this study, the average age of the children in the treatment and nontreatment groups was 5.04 years and 4.38 years, respectively. The *χ^2^* test revealed no significant difference in age (*χ^2^*_3_=7.38, *P*=.06) between the two groups. We divided the children into four age groups and determined whether the children in these four age groups had different injection experiences, expected pain before injection, pain-related response during injection, and actual pain. The Kruskal-Wallis test revealed no significant age-based differences in either group. The child participants in our study were limited to hospitalized children aged 3-7 years; therefore, our results cannot fully indicate whether age was an influencing factor. Future studies could explore this issue.

Some scholars have reported that sex does not affect the pain levels felt by children [22,30,34], whereas others have reported that girls experience greater levels of pain [23,35], revealing inconsistency regarding the effect of sex on pain level. In our study, the treatment group included more boys (n=19, 63%), whereas the nontreatment group had more girls (n=18, 60%); the *χ^2^* test showed no significant difference between the two groups (*χ^2^*_1_=3.27, *P*=.07). The Mann-Whitney *U* test was performed to determine whether children of different sexes had differing injection experiences, pain-related response during injection, and actual pain. In the nontreatment group, injection experience and pain-related response during injection were significantly different between the boys and girls (*Z*=–2.441, *P*=.02; *Z*=–2.566, *P*=.01), whereas in the treatment group, significance was not achieved in any of these three aspects. This result showed that the use of Buzzy closed the gap between the sexes regarding pain level. This result is consistent with that of another study in which an intervening measure was employed to reduce pain [22].

### Implementation

#### Clinical Practice

The use of Buzzy is noninvasive and can be employed independently by nurses without medical advice. Before use, a simple assessment of suitability was performed in this study, and the device was then illustrated and its operation explained to the children. This obtained their trust and enhanced their cooperation during the process to achieve maximum pain relief. The Buzzy device is worthy of consideration and application by nurses. The device has a cute appearance; nurses could integrate it into therapy-related games by giving it a human voice, which is often effective with hospitalized children. Buzzy can also be considered for use during other invasive procedures such as intramuscular injection and blood sugar measurement.

According to our results, measures for relieving pain during intravenous injection should have certain characteristics, including being suitable for most children, easy to use, having a short preparation time, and not affecting the rate of successful injection or procedural duration; additionally, no discomfort or injury should occur as a result of using the tool. Pain relief during injections should be proactively provided and routinely included in procedures. Use of an intervening measure enhances the emotional preparedness of the child and in turn enhances the measure’s acceptability. Effective pain relief results in a satisfactory injection experience and prevents unpleasant experiences from affecting every injection, thereby enhancing the quality of care and building a high-quality nurse-patient relationship.

#### Future Research

This study did not find age-related differences. In the absence of the pain relief measure, the girls reported a poorer injection experience and greater pain than the boys, but no significant difference was determined between the sexes when Buzzy was used. However, our participants were recruited on the basis of order of admission and need for injection; thus, the research design could not control or ensure an equal number of cases for each age group and sex. These two influencing factors should be considered in further exploration. In the future, researchers could consider controlling for age and sex. For future application, researchers could integrate the Buzzy device into a technology-based app for increasing the efficiency of use and provide more data collection functions.

#### Policy

From this study, we conclude the need to consider using pain relief measures during intravenous injection in children during routine nursing procedures. Additionally, adequate equipment should be provided and relevant in-service education and experience-sharing organized to ensure the capability of clinical staff in equipment operation. An example of an effective measure is the Buzzy device, which was used in this study. Legitimate devices should be obtained through official channels, and users must pay attention to the safety of the device. The device should not be used on patients with paresthesia or at an injection site that has broken skin. Usage guidelines and indications must be formulated for the device, including those regarding the principle of the cold sensation, device disinfection, and regular maintenance. Attention must be paid to individual differences among children to ensure that a device or measure is suitable for a given child.

### Limitations and Recommendations

Surgical operations can affect pain assessment, and children with cognitive impairment cannot adequately and correctly express themselves; thus, we did not include children with these conditions. Our results cannot be extrapolated to these populations. Additionally, studies have indicated that fear is lower when pain relief is satisfactory [13,36], revealing that pain and fear affect each other. The age group of our participants was preschool children. This study used the WBFS to measure pain because children may be confused about their feelings and the WBFS is a clear and simple measurement method. However, this study only measured pain, and the degree of fear of the participants could not be inferred. Further research should be conducted on this aspect.

We recruited participants from only one medical center because of time and human resource considerations. However, the medical treatment of children varies according to region and institution habits, which could lead to differences in demographics, previous injection experiences, and present injection experiences. Additionally, our study did not employ random sampling, and therefore the results cannot be extrapolated to the total hospitalized child population of Taiwan. We recommend performing a controlled experiment with random group allocation if recruitment is easy and the sample is large. Moreover, to determine whether sex affects pain level, we recommend employing sex as a control variable in analyses to reduce errors. The age range of participants should be expanded to elucidate whether needle pain differs with age.

### Conclusion

The participants of our study were hospitalized children. Most of these children had experience of intravenous injections and expected to feel pain. In the present medical environment in Taiwan, most clinical institutions do not have time to instruct and console patients, which could reduce needle pain. Our study discovered that most of the children had unpleasant experiences of injection, and because an intervening measure was not employed, the children felt moderate or severe pain during intravenous injection. Most of the children and parents wished for an effective pain relief measure. In our study, the Buzzy device effectively reduced needle pain in the children; the pain-related response during injection (FLACC score) and actual reported pain of the treatment group were significantly different from those of the nontreatment group. The pain-related response of the children during injection was reduced, indicating a satisfactory experience.

The use of Buzzy in our study did not affect the rate of successful injection or the injection duration; this result could increase the willingness of clinical staff to use the device and boost the utilization of pain relief measures during injections to prevent children from having negative experiences. We also discovered that although some children had unpleasant injection experiences, Buzzy could still reduce needle pain. Effective pain relief measures during intravenous injections should be routinely administered.

Our research participants were limited to hospitalized children aged 3-7 years. Although we could not determine whether age was a factor affecting pain level, the use of Buzzy reduced the degree of pain to the same degree for boys and girls. Researchers could use this result as a reference when selecting research participants in Asian areas, as well as when considering the influencing factors in future research.

## Abbreviations

FLACC: Faces, Legs, Activity, Cry, Consolability
WBFS: Wong-Baker Face Scale
